# A75 PEDIATRIC ABDOMINAL PAIN PRESENTING TO A HOSPITAL-BASED GI CLINIC: EVOLUTION AND DISPOSITION

**DOI:** 10.1093/jcag/gwad061.075

**Published:** 2024-02-14

**Authors:** H Brill, R Patel

**Affiliations:** Pediatrics, University of Toronto, Toronto, ON, Canada; University of Ottawa, Ottawa, ON, Canada

## Abstract

**Background:**

Abdominal pain accounts for 50% of pediatric gastroenterology consultations. While data exists on the possible etiologies of abdominal pain, there is a paucity of data on the natural evolution of abdominal pain while under the care of a Pediatric Gastroenterologist. Given limited access to Pediatric Gastroenterologists and long waiting times for initial consultation for abdominal pain, understanding what interventions ameliorate pain may help Gastroenterologists advise referring physicians while the patient awaits a consultation.

**Aims:**

Primary outcome measure was the percentage of patients who reported at least a 75% subjective improvement of pain over baseline consultation.

**Methods:**

A retrospective chart review between April 2014 and Dec 31, 2022 was taken in a hospital based GI clinic. Subjects referred for assessment of abdominal pain were identified, along with subjects referred for reflux and dysphagia as a comparator group. Subsequent visits were abstracted to identify diagnosis disposition and assess which interventions were tried and to what extent they succeeded. Patient loss to follow up were also measured. Results were summarized using descriptive statistics, and regression modeling will be attempted to identify predictors of response.

**Results:**

393 subjects were referred for abdominal pain and 286 for GERD and dysphagia. 439 (64.7%) reported at least 75% improvement in symptoms. 242 (35.6%) underwent endoscopy, 263 (38.7%) used Proton Pump Inhibitors, and 38 (8.54%) used Polyethylene Glycol-3350. Carbohydrate eliminations of various types were used in 13.5 - 27% of subjects with pain. 193 subjects (28.5%) were ultimately lost to follow up though some did report improvement in symptoms. Only 45 (6.6%) remained in active care by the end of the study while only 13 (1.93%) were transitioned to an Adult Gastroenterologist at 18 years old.

**Conclusions:**

Pediatric Abdominal Pain is primarily a transitory population in the Pediatric GI clinic, with a large number responding to successive interventions. There is also a sizable minority who are lost to follow up. Future studies should attempt to identify the reasons for loss to follow up and ultimate disposition.

**Funding Agencies:**

William Osler Health System Summer Student Research Program

